# Supplementary material for: Differential Roles of Five Fluffy Genes (flbA–flbE) in the Lifecycle In Vitro and In Vivo of the Insect–Pathogenic Fungus Beauveria bassiana
Source: J Fungi (Basel). 2022 Mar 23;8(4):334. doi: 10.3390/jof8040334 (PMC9031332; doi:10.3390/jof8040334)
Supplement: Supplementary file 1 [file jof-08-00334-s001.zip › jof-1590719-supplementary.pdf]

Supplementary Materials for *Journal of Fungi*

Differential roles of five fluffy genes (*flbA–flbE*) in the lifecycle *in vitro* and *in vivo* of the insect-pathogenic fungus *Beauveria bassiana*

Chong-Tao Guo, Xin-Cheng Luo, Sheng-Hua Ying, and Ming-Guang Feng*

Institute of Microbiology, College of Life Sciences, Zhejiang University, Hangzhou, Zhejiang, 310058, China
*Correspondence to Ming-Guang Feng, mgfeng@zju.edu.cn

Table S1. Paired primers used for manipulation of *flbA, flbB, flbC, flbD and flbE* in *B. bassoama*.

Primers	Paired sequences (5′–3′)*	Purpose
FlbAc-F/R	CACAAACACCTTCAAACCCGGGATGGCAGCTTTGTTGAACCA / TGCTCACCATGTTAACGGATCCATTTCGATTCGATCGGCTTT	Cloning *flbA* cDNA (2064 bp) for fusion to *GFP*
FlbBc-F/R	CACAAACACCTTCAAACCCGGGATGTCTGCCTCGTTGTCTCC / TGCTCACCATGTTAACGGATCCATACGCCATTTGCTCCAATC	Cloning *flbB* cDNA (1092 bp) for fusion to *GFP*
FlbCc-F/R	CACAAACACCTTCAAACCCGGGATGACCATGGCTTTGGATCCT / TGCTCACCATGTTAACGGATCCATCCGAGTGGTGCTCTTCG	Cloning *flbC* cDNA (1125 bp) for fusion to *GFP*
FlbDc-F/R	CACAAACACCTTCAAACCCGGGATGGAAGAAGATGCTACAGCC / TGCTCACCATGTTAACGGATCCGTGCAGTAGAGACGATACGGAA	Cloning *flbD* cDNA (813 bp) for fusion to *GFP*
FlbAup-F/R	GTACAAGTAACCCGGGGATCCGTGCTGAGGGCATTGAGAA / ACCGAGATTTGACCATGGGAGCTCGGGACAAGACAAGAAGCGTAA	Cloning *flbA* 5′-end (1503 bp) for targeted gene deletion
FlbAdn-F/R	GTCGACCCATGGCTCGAGTCTAGAAGCCCCTGTAGTTTTGTGTCC / GGTGGTGGTGGCTAGCGTTAACCTGTCTCTCCTCCGCTCTCC	Cloning *flbA* 3′-end (1532 bp) for targeted gene deletion
FlbAfl-F/R	*GGGGACAAGTTTGTACAAAAAAGCAGGCT*ATCACGCACACAATCCAGTAAT / *GGGGACCACTTTGTACAAGAAAGCTGGGT*CGGGTAGTAGGCACCAACAT	Cloning full-length *flbA* (5176 bp) for *flbA* complementation
FlbBup-F/R	GTACAAGTAACCCGGGGATCCCCGTTCCCTGGTGTGTGTC / ACCGAGATTTGACCATGGGAGCTCAACCAGATACTGCGGCACTAAT	Cloning *flbB* 5′-end (1528 bp) for targeted gene deletion
FlbBdn-F/R	GTCGACCCATGGCTCGAGTCTAGAGCTGTGATGAACAAAGCAAAC / GGTGGTGGTGGCTAGCGTTAACCGTCTCGCTCTCGGTCAC	Cloning *flbB* 3′-end (1488 bp) for targeted gene deletion
FlbBfl-F/R	*GGGGACAAGTTTGTACAAAAAAGCAGGCT*ATGGCGTTGATTGAAAGAGTA / *GGGGACCACTTTGTACAAGAAAGCTGGGT*GCAGATTTTCCCGTAGTCG	Cloning full-length *flbB* (4115 bp) for *flbB* complementation
FlbCup-F/R	GTACAAGTAACCCGGGGATCCACTGGCAATCTGTAGACCTCC / ACCGAGATTTGACCATGGGAGCTCCGCCGCAATGTCATCAAC	Cloning *flbC* 5′-end (1574 bp) for targeted gene deletion
FlbCdn-F/R	GTCGACCCATGGCTCGAGTCTAGAGGCGTTTATGGTTACTGGGG / GGTGGTGGTGGCTAGCGTTAACCGCATACCAACAGTCGTCCT	Cloning *flbC* 3′-end (1472 bp) for targeted gene deletion
FlbCfl-F/R	*GGGGACAAGTTTGTACAAAAAAGCAGGCT*TACCCGCTACGAATGAACAC / *GGGGACCACTTTGTACAAGAAAGCTGGGT*TATCTCACCCAAAACTAAAGCC	Cloning full-length *flbC* (3765 bp) for *flbC* complementation
FlbDup-F/R	GTACAAGTAACCCGGGGATCCATTTACCGATGTTGCTCAGAAG / ACCGAGATTTGACCATGGGAGCTCAGATTTGGAAGGGCAGGGT	Cloning *flbD* 5′-end (1584 bp) for targeted gene deletion
FlbDdn-F/R	GTCGACCCATGGCTCGAGTCTAGATATTGAGAGAGGCTCGGACC / GGTGGTGGTGGCTAGCGTTAACCGTTTTCCCCTTCAGCAG	Cloning *flbD* 3′-end (1554 bp) for targeted gene deletion
FlbDfl-F/R	*GGGGACAAGTTTGTACAAAAAAGCAGGCT*ATACTTTGGCTTCTTCGGTTTC / *GGGGACCACTTTGTACAAGAAAGCTGGGT*TGCTTCTTCTATCTGCGTGCTA	Cloning full-length *flbD* (3266 bp) for *flbD* complementation
FlbEup-F/R	GTACAAGTAACCCGGGGATCCATCCATCCGCTGATTCCGTTTTTAT / ACCGAGATTTGACCATGGGAGCTCACGGGCTGAGTTGAAAGGAA	Cloning *flbE* 5′-end (1563 bp) for targeted gene deletion
FlbEdn-F/R	GTCGACCCATGGCTCGAGTCTAGAGCGAAGAAAGATGTCAGTGGC / GGTGGTGTGGCTAGCGTTAACGGGGGTTTGTCTGGAGGTC	Cloning *flbE* 3′-end (1455 bp) for targeted gene deletion
FlbEfl-F/R	*GGGGACAAGTTTGTACAAAAAAGCAGGCT*CCGTTCGGAAGAGGGATAA / *GGGGACCACTTTGTACAAGAAAGCTGGGT*TCGCCGTCTCACAGCATAG	Cloning full-length *flbE* (3824 bp) for *flbE* complementation
pFlbA-F/R	ACTGGTCTTGAACCGTTTTTG / GGGCGTCGTGTCTAAGAACTA	PCR detecting *flbA*
pFlbB-F/R	CGCTGCCTACTTGAGACGAC / CGGAATACGGTTTTGTGACTG	PCR detecting *flbB*
pFlbC-F/R	ATTACCGCCATTACTACTATCTGAA/ AACCAAACAATGAAGCCAAAT	PCR detecting *flbC*
pFlbD-F/R	AATAGAGGCGTCAGAATAGCAAAG/ AAAAAACCCTGTTCGCCTTG	PCR detecting *flbD*
pFlbE-F/R	ATGTGGAGGTCGTCGGAAAG/ ACTCAGAAGAGGAATCAAAAAGACA	PCR detecting *flbE*
qFlbA-F/R	CCATCACGAAAACGATCGGC / TCTTTGGCGCGAGACAGAAT	qPCR detecting *flbA*
qFlbB-F/R	AGATTCCGAGTCAGGGGACA / CTTTCAAAAGCGTCCCGCAA	qPCR detecting *flbB*
qFlbC-F/R	TCTCTAACCCGTGGTCGTCT / CCAAGGGCATAGAGCCGTAG	qPCR detecting *flbC*
qFlbD-F/R	TCGAACTGCCAACCTCTCAC / TTGAACCGGAGAGTTCGGTG	qPCR detecting *flbD*
qFlbE-F/R	AGAATGCCTGGTTCTCGACG / GCTGCTGTTTGTGGTCAAGG	qPCR detecting *flbE*
qActin-F/R	GGCAACATTGTCATGTCTGG /TTTGCTGGAAGGTGGATAGG	qPCR detecting β-actin gene

* The underlined regions denote the restriction enzyme sites for the fusion of *flbA*, *flbB*, *flbC* or *flbD* cDNA (*Xma*I/*Bam*HI) fusion to *GFP* and the deletion of each *flb* gene (*BamH*I/*Sac*I and *Xba*I/*Hpa*I). The underlined and italicized regions are the recognition fragments to exchange for the gateway fragment in complementary plasmid.

Table S2. Paired primers used in the qPCR analysis of phenotype-related genes in *B. bassiana.*

Gene	Tag loci*	Annotation	Sequences (5′-3′) of paired primers
**Involved in asexual development**			
brlA	BBA_07544	Key CDP activator BrlA	CGGCCGTTACTACATCCAGG / ACTCCGTTTCCATCGCACTT
abaA	BBA_00300	Key CDP activator AbaA	CCACGGCATGAACCTGTTTG / CGTTCGAGGGCAAAAAGTGG
**Involved in conidial hydrophobicity and adherence to insect cuticle**			
hyd1	BBA_03015	class I hydrophobin Hyd1	GTACCTGCACCAAGATCCCC / AAGACCACCGTAAGCGACAG
hyd2	BBA_06599	Class II hydrophobin Hyd2	CCAGCGTTCTCAGCGATCTTCTGCTGCTTGCACTTGTTGT
hyd3	BBA_00530	Hydrophobin-like protein Hyd3	CGGCGACGACTTTAAGGGAA / CTAGGAGAATTGCCGCCTGT
hyd4	BBA_03071	Hydrophobin-like protein Hyd4	TACTGAGCTTGCTCCCCGTA / AGCAACACCGAGGATGTCAG
hyd5	BBA_02999	Hydrophobin-like protein Hyd5	TGCTATCGCTGCTCTCTTCG / AGGTTGGAGTAGAGACCGCT
**Involved in cuticle degradation and host infection**			
pr1A1	BBA_04506	Subtilisin-like protease Pr1A1	TTCCGTACCTCGGACCTGAT / CGAGAAGTATCTCGCGTCCC
pr1A2	BBA_08580	Subtilisin-like protease Pr1A2	CGGCGAGACCAAGGACATTA / CTTCCGAGTAGACGGTGTCG
pr1B1	BBA_03653	Subtilisin-like protease Pr1B1	TTGCGTACTGCAAGCGAAAG / AACTCTGCTTTGTCCAGCGA
pr1B2	BBA_00443	Subtilisin-like protease Pr1B2	CTACCTTTTGGCTCTCGGCA / ATGCCAGGTAGTTGACGGTG
pr1B3	BBA_04617	Subtilisin-like protease Pr1B3	CTCCTCACATTGCCGGTCTT / AAGCAGACCAGTGCTGACTC
pr1C	BBA_09153	Subtilisin-like serine protease Pr1C	ATATCTGGCCCGTCAAGCTG / GTCGCGGGAGTCGATAATGT
pr1F1	BBA_10157	Subtilisin-like protease Pr1F1	CTCATCGGGAGAAACCCAGG / TCTCAGCACGACCCTCAAAC
pr1F2	BBA_07320	Subtilisin-like protease P11F2	CTACTACGCTTCTGGGGCTG / GGCATACTTGGGCTTTTCGC
pr1F3	BBA_09501	Subtilisin-like protease Pr1F3	ACTTGGGCGAGAGCTAACAC / AGGTCTTGGAGGCAATCGTG
pr1F4	BBA_07143	Subtilisin-like protease Pr1F4	CGGCGGATTATTCGTACCCA/TGGCCACTCCGTAGGTTTTC
pr1G	BBA_09270	Subtilisin-like protease Pr1G	TAGGCGGCTCCTTTTCACAG / TGGGACCCTTTTTCTCGTCG
**Involved in heat shock**			
hsp20	BBA_07886	Heat shock protein Hsp20	TCGACCTCTTTAACACGCCC / GAGTGCAGCGACTGCAAAAA
hsp30a	BBA_08688	Heat shock protein Hsp30a	CGAATTCCCGGAACCACAGA / TGATGGTTGGCTTGTCCGAA
hsp30b	BBA_02057	Heat shock protein Hsp30b	ATGACGTGAGCGTCGAGTTT / GTCCTCATCAGACACGGTGG
hsp40a	BBA_06930	Heat shock protein Hsp40a, DnaJ	CAGCCAAGTCATCCAGGGTT / TCTCGGAAGCGGATGCATTT
hsp40b	BBA_08440	Heat shock protein Hsp40b, DnaJ	ACATGTCGAGACAGTGGCTG / TGCTTCTTGCGCCAAGTTTC
hsp40c	BBA_03736	Heat shock protein Hsp40c, DnaJ	GACCAGTTCCCCGACCAAAT / GCCTCGCTGAGGGAAATCTT
hsp60	BBA_05467	Heat shock protein Hsp60	GCATTACTCCCCAATGGGCT / TGTCTGCATGTCACGCTCTT
hsp70a	BBA_00941	Heat shock protein Hsp70a	AGAATGCGTCCCTGGTGAAG / TGGTCGACAAAGATGCCTCC
hsp70b	BBA_05586	Heat shock protein Hsp70b	AGGAGCCCAACAAGAGCATC / ATGGTGGTGTTGCGAGGAAT
hsp90	BBA_06516	Heat shock protein Hsp90	GGTGCCGATGTCTCCATGAT / TCGTCATCGTTGTGCTTGGA
hsp104	BBA_02283	Heat shock protein Hsp104	AGAGTTTCTCACGCGTCCTG / ATGCGGTTCAGGAACTCTGG
**Involved in response to oxidative stress**			
sod1	BBA_02311	Cytocolic Cu/Zn-SOD Sod1	ACATTAAGACGGACGCCCAG / TGCCAGTCTTGAGGGACTCT
sod2	BBA_09706	Cytosolic Mn-SOD Sod2	CCCTACGCCTACGATGCTTT / AGAGCGAGTGGTTGATGTGG
sod3	BBA_09382	Mitochondrial Mn-SOD Sod3	GCCAACCAAGATCCCATTGC / ATTCAGCCTTGCGGTTCTCA
sod4	BBA_04317	Mitochondrial Fe-SOD Sod4	AGCTTCCTTTTCCTCCGTCG / ACGGTGTTCATGTCGGTTGA
sod5	BBA_01984	Cell wall-anchored Cu/Zn-SOD Sod5	TCACCGACCCTTTCACTTCG / TCAACCTTCTGGAAGTCGGC
cat1	BBA_06186	Spore-specific catalase CatA	TTGCGTTTTGCACTAGCCAC / AGCTCTTCCCAGTTGATGCC
Cat2	BBA_05603	Secreted catalase CatB	GAGTCGGTCGATGCTACCAG / CCGCCTTTAGGCTTTCCTGA
Cat3	BBA_09109	Cytoplasmic catalase CatC	GCTACGGAAACACGAATGGC / CAGACACATCGTCCAGCACT
Cat4	BBA_09760	Secreted catalase CatD	CTTTTTGTGTACGCCGGCAA / ATGTCTGTGCTGGCGTTGTA
Cat5	BBA_09338	Peroxisomal catalase CatP	AGATTGCCGTCTCCCACAAG / GTCGGGGTTCTTTCTACCCG
cat6	BBA_06567	Catalase-like domain, heme-dependent	TGGAAAGAGACGATTGCGCT / TTTACCCGGGCAACGATTCA

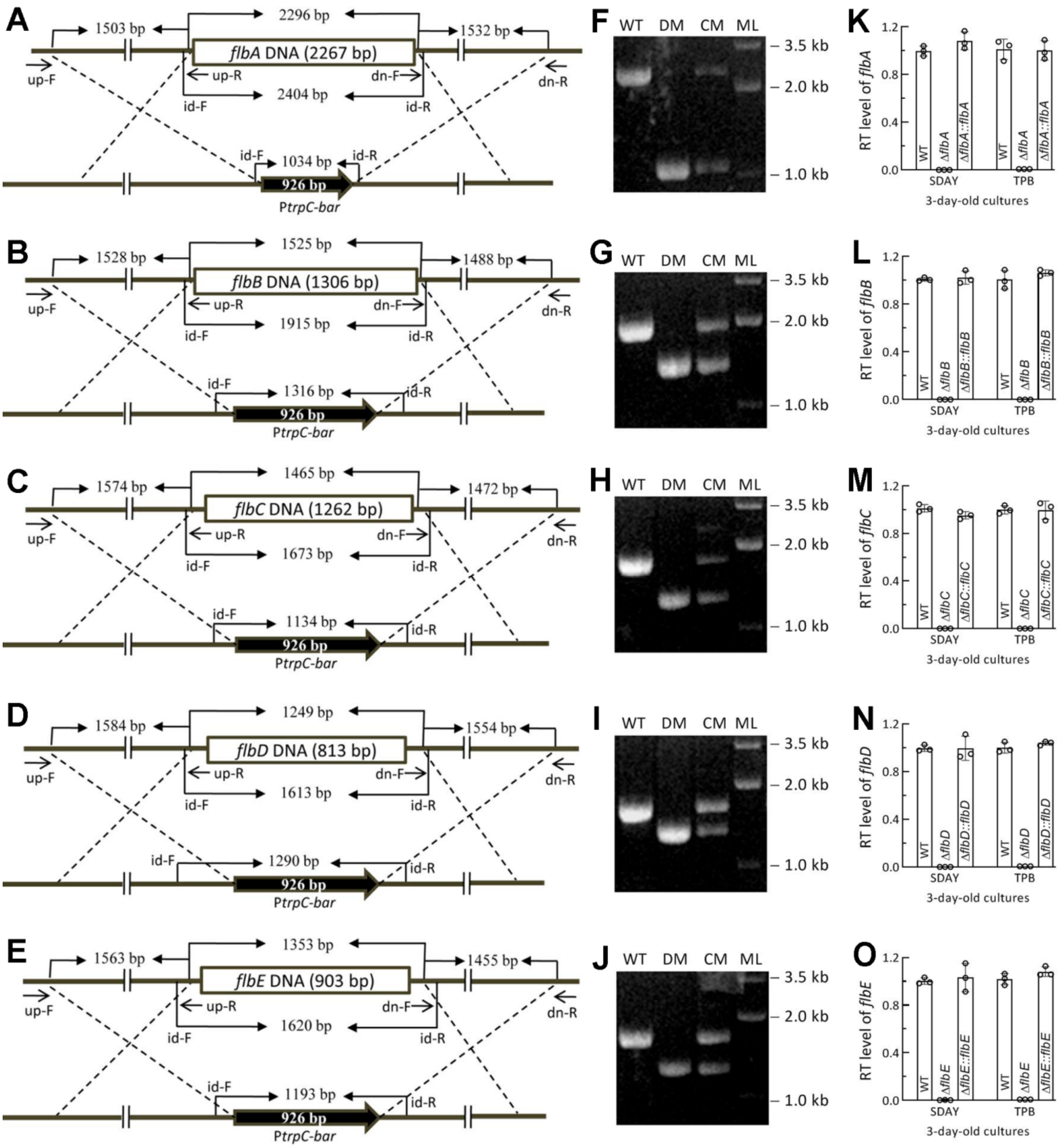

Figure S1. Generation and identification of *flbA*–*flbE* mutants in *B. bassiana*. (**A–E**) Diagrams for single-gene deletion strategies of *flbA*–*flbE*. (**F–J**) The *flbA*, *flbB*, *flbC*, *flbD* and *flbE* mutants identified via PCR analysis with paired primers (see Table S1), respectively. Indicated by the detected DNA fragments, the full-length coding and partial flanking regions of each target gene were successfully deleted from the WT strain as expected (2404 + 926 – 1034 = 2296 bp for *flbA*, 1915 + 926 – 1316 = 1525 bp for *flbB*, 1915 + 926 – 1316 = 1525 bp for *flbC*, 1613 + 926 – 1290 = 1249 bp for *flbD*, and 1620 + 926 – 1193 = 1353 bp for *flbE*). DM, deletion mutant. CM, complementation mutant. ML, molecular ladder of genomic DNA. (**K–O**) Relative transcript (RT) levels of *flbA*, *flbB*, *flbC*, *flbD* and *flbE* in the 3-d-old SDAY and TPB cultures of their deletion and complementation mutants with respect to the WT standard. Note that the expression of *each flb gene* was not detectable in DM but well restored by targeted gene complementation. Error bars: standard deviations of the means from three cDNA samples derived from independent cultures of each strain.

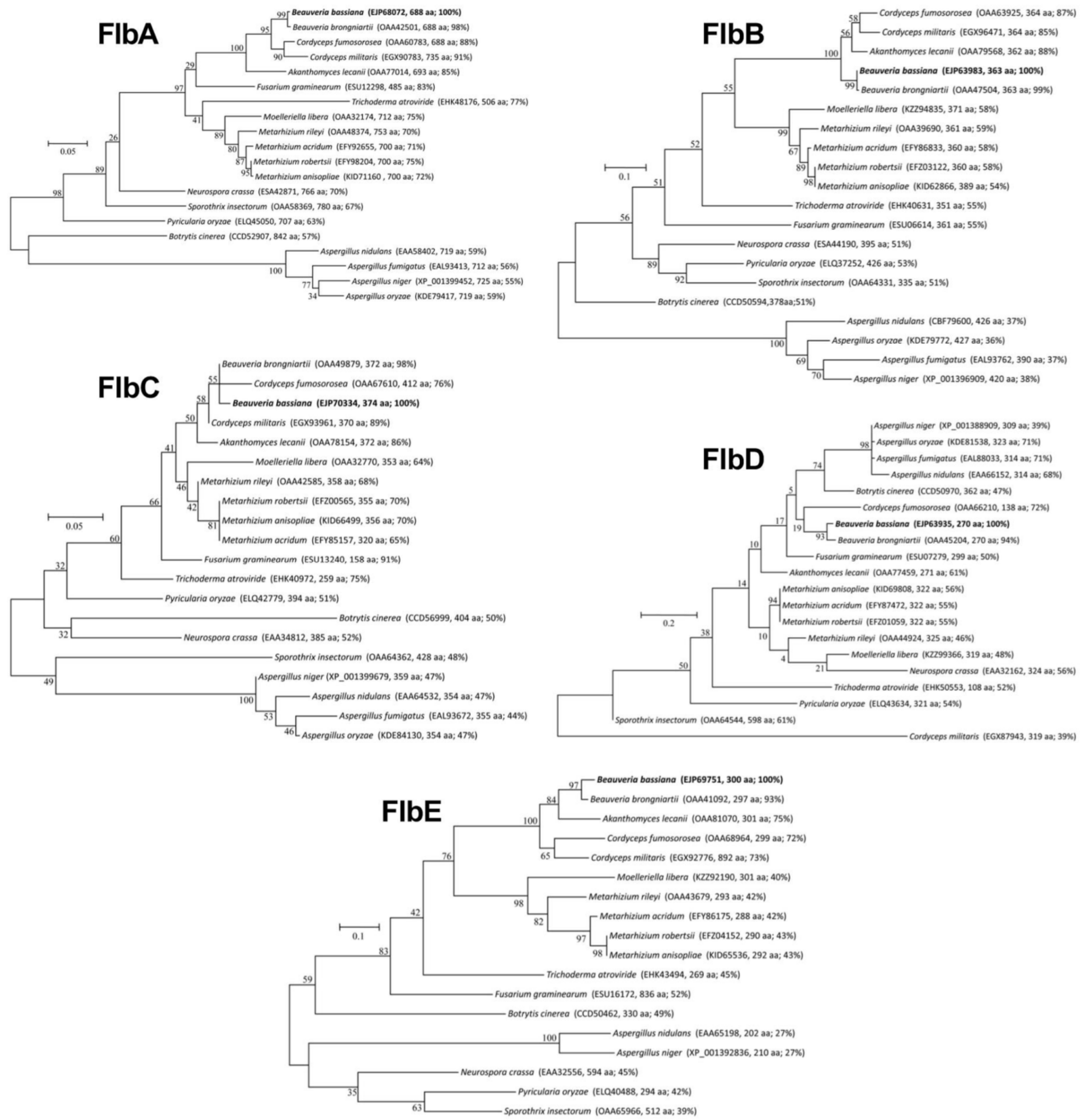

Figure S2. Phylogenetic trees of FlbA–FlbE orthologues found in selected filamentous fungi. Each tree was constructed with the maximum likelihood method in MEGA7 at http://www.megasoftware.net/ (accessed on 23 March 2022). Bootstrap values of 1000 replications are shown at nodes. Scale: branch length proportional to genetic distance. The NCBI accession code of each protein, the length of its amino acid sequence, and its protein sequence identity to its orthologue (in bold) in *B. bassiana* are given in the parentheses following the fungal name.

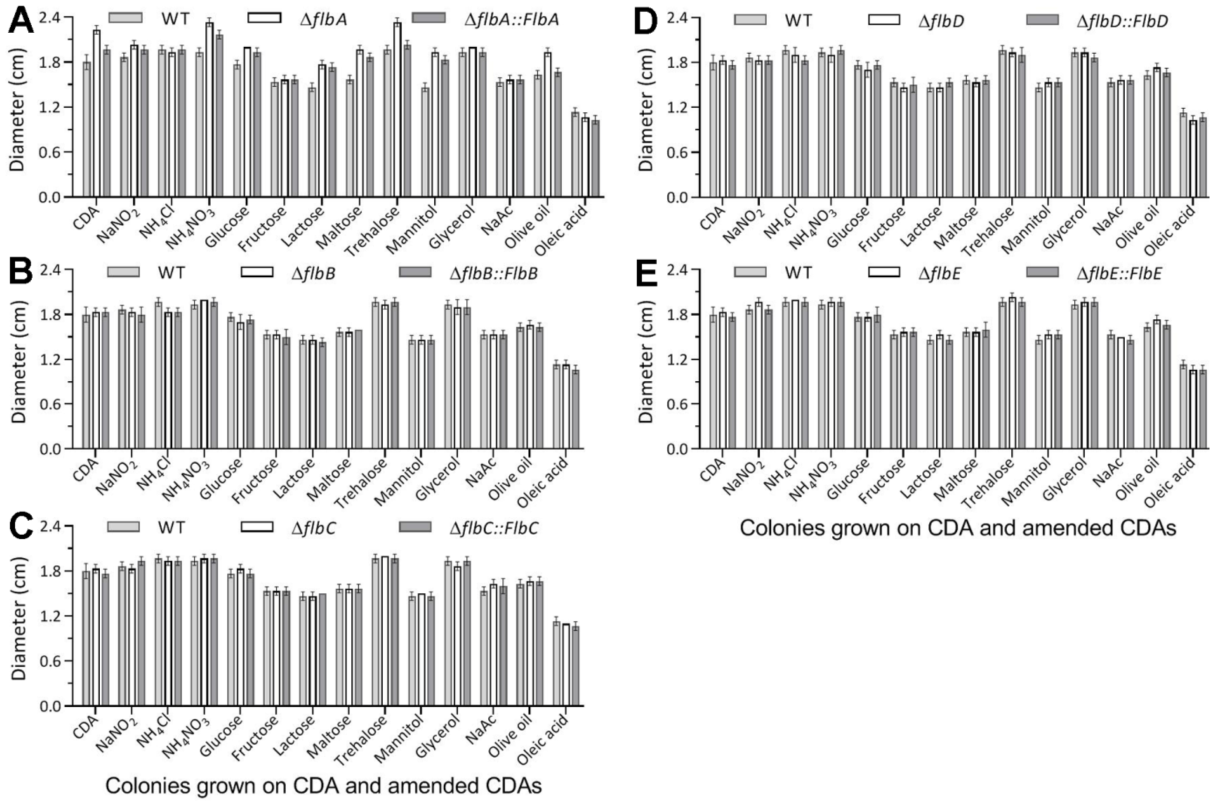

Figure S3. The diameters of fungal colonies grown at the optimal regime of 25°C and L:D 12:12 for 7 d on the plates of minimal CDA and CDAs amended with different carbon or nitrogen sources. Each colony was initiated by spotting 1 μL of a 10^6 conidia/mL suspension. Error bars: standard deviations of the means from three independent replicates.

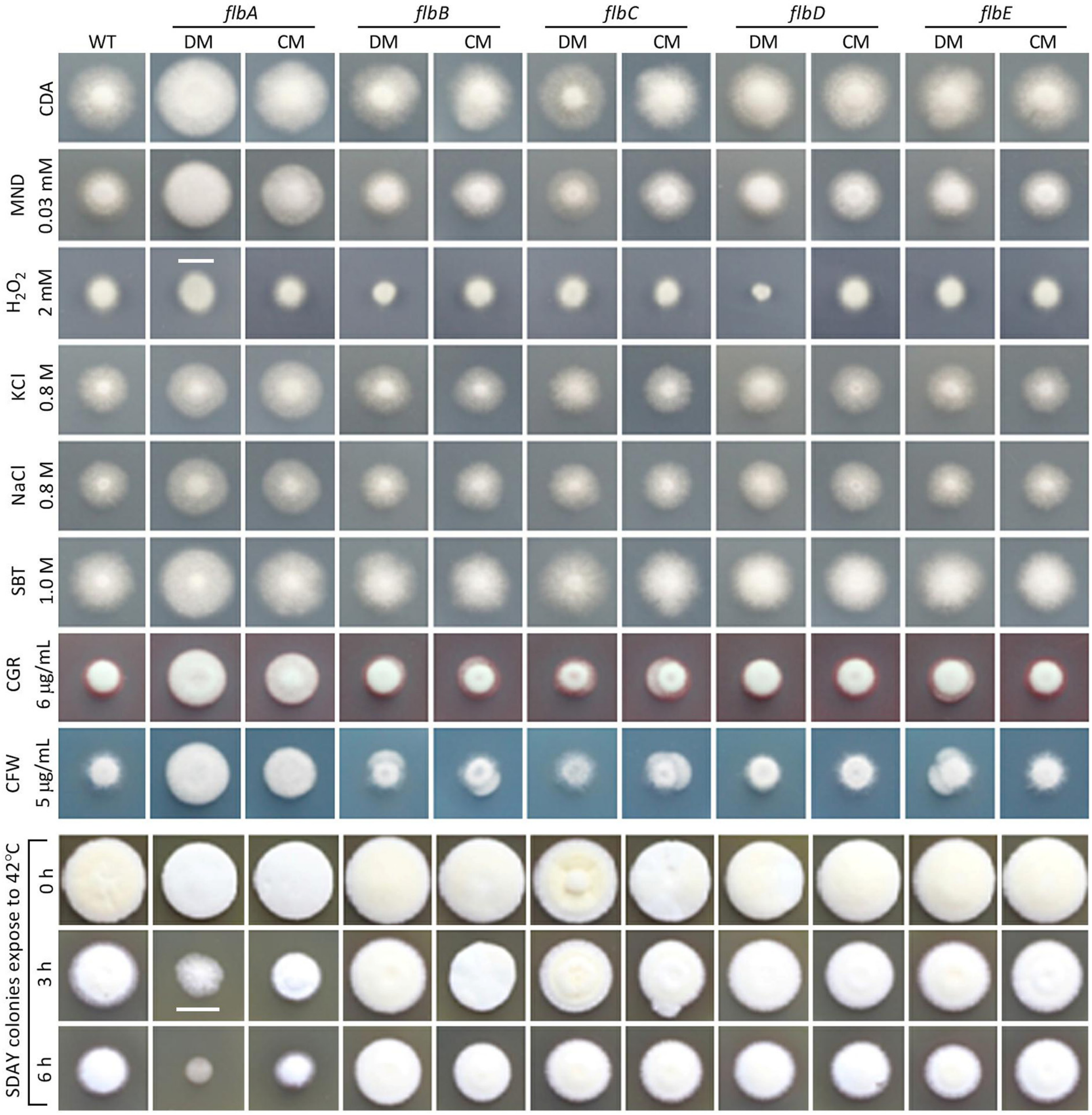

Figure S4. Images (scale: 10 mm) of fungal colonies incubated at 25°C for 7 d on the plates of CDA alone (control) or supplemented with the indicated concentrations of menadione (MND), H_2O_2, KCl, NaCl, sorbitol (SBT), Congo red (CGR) and calcofluor white, respectively, and of SDAY colonies incubated at 25°C for 5-d growth recovery after 2 d-old colonies were exposed to a 42°C heat shock for 3 and 6 h. Each colony was initiated by spotting 1 μL of a 10^6 conidia/mL suspension.
